# Citric Acid Cycle Metabolites Predict Infarct Size in *Pigs* Submitted to Transient Coronary Artery Occlusion and Treated with Succinate Dehydrogenase Inhibitors or Remote Ischemic Perconditioning

**DOI:** 10.3390/ijms22084151

**Published:** 2021-04-16

**Authors:** Marta Consegal, Norberto Núñez, Ignasi Barba, Begoña Benito, Marisol Ruiz-Meana, Javier Inserte, Ignacio Ferreira-González, Antonio Rodríguez-Sinovas

**Affiliations:** 1 Cardiovascular Diseases Research Group, Department of Cardiology, Vall d’Hebron Institut de Recerca (VHIR), Vall d’Hebron Hospital Universitari, Vall d’Hebron Barcelona Hospital Campus, Passeig Vall d’Hebron 119-129, 08035 Barcelona, Spain; marta.consegal@vhir.org (M.C.); ignasibarba@gmail.com (I.B.); begona.benito@vhir.org (B.B.); mruizmeana@gmail.com (M.R.-M.); javier.inserte@vhir.org (J.I.); 2 Departament de Medicina, Universitat Autònoma de Barcelona, 08193 Bellaterra, Spain; 3 Centro de Investigación Biomédica en Red (CIBER) de Enfermedades Cardiovasculares (CIBERCV), Instituto de Salud Carlos III, 28029 Madrid, Spain; 4 Unit of High Technology, Vall d’Hebron Institut de Recerca (VHIR), Vall d’Hebron Hospital Universitari, Vall d’Hebron Barcelona Hospital Campus, Passeig Vall d’Hebron 119-129, 08035 Barcelona, Spain; norberto.nunez@vhir.org; 5 Faculty of Medicine, University of Vic-Central University of Catalonia (UVicUCC), Can Baumann. Ctra. de Roda, 70, 08500 Vic, Spain; 6 Centro de Investigación Biomédica en Red (CIBER) de Epidemiología y Salud Pública (CIBERESP), Instituto de Salud Carlos III, 28029 Madrid, Spain

**Keywords:** succinate dehydrogenase, malonate, remote ischemic conditioning, myocardial infarction, ischemia-reperfusion

## Abstract

Succinate dehydrogenase (SDH) inhibition with malonate during reperfusion reduced myocardial infarction in animals, whereas its endogenous substrate, succinate, is detected in plasma from STEMI patients. We investigated whether protection by SDH inhibition is additive to that of remote ischemic perconditioning (RIC) in *pigs* submitted to transient coronary artery occlusion, and whether protective maneuvers influence plasma levels of citric acid cycle metabolites. Forty *pigs* were submitted to 40 min coronary occlusion and reperfusion, and allocated to four groups (controls, sodium malonate 10 mmol/L, RIC, and malonate + RIC). Plasma was obtained from femoral and great cardiac veins and analyzed by LC-MS/MS. Malonate, RIC, and malonate + RIC reduced infarct size (24.67 ± 5.98, 25.29 ± 3.92 and 29.83 ± 4.62% vs. 46.47 ± 4.49% in controls, *p* < 0.05), but no additive effects were detected. Enhanced concentrations of succinate, fumarate, malate and citrate were observed in controls during initial reperfusion in the great cardiac vein, and most were reduced by cardioprotective maneuvers. Concentrations of succinate, fumarate, and malate significantly correlated with infarct size. In conclusion, despite the combination of SDH inhibition during reperfusion and RIC did not result in additive protection, plasma concentrations of selected citric acid cycle metabolites are attenuated by protective maneuvers, correlate with irreversible injury, and might become a prognosis tool in STEMI patients.

## 1. Introduction

Early diagnosis and immediate application of reperfusion therapies are the most effective ways to preserve viability of the ischemic myocardium and limit infarct size in patients with ST-segment elevation myocardial infarction (STEMI). Preventive measures and advances in reperfusion therapies have greatly contributed to the reductions in mortality and morbidity observed in STEMI patients in the last decades [1]. However, the risk of cardiovascular events has been shown to remain high beyond the first year post-myocardial infarction [2]. Despite appropriate and timely application of reperfusion therapies, a high proportion of STEMI patients end up with extensive areas of myocardial necrosis, that would compromise cardiac function, leading to heart failure and arrhythmias, and eventually death. This is in part due to the existence of reperfusion injury triggered by blood flow restoration itself, a phenomenon consisting of an additional cell death to that induced by ischemia [3]. Indeed, it has been proposed that lethal reperfusion injury may account for about half of the final infarct size [4]. However, the discovery of reperfusion injury opened a window of opportunity to explore new therapeutic approaches to reduce final infarct size. Experimental studies have allowed us to identify a number of strategies able to attenuate reperfusion injury in different animal models [5]. Nevertheless, none of these studies have become part of standard clinical care. This is probably due to their limited protective effects in STEMI patients, which are often influenced by comorbidities, concomitant medications, and other factors, such as age [6]. Thus, further efforts are needed to identify new strategies that are able to mitigate reperfusion injury in the clinical setting.

Among these is the inhibition of mitochondrial succinate dehydrogenase (SDH) [7,8,9]. SDH or mitochondrial respiratory chain complex II is the enzyme that catalyzes oxidation of succinate to fumarate within the citric acid cycle, in a process that results in the donation of electrons to the mitochondrial respiratory chain via reduction of FAD to FADH_2_. However, under some circumstances, SDH may work in the reverse direction, reducing fumarate and leading to succinate accumulation [9,10,11]. In this regard, previous studies have demonstrated that succinate accumulates in ischemic tissues due to the reversal action of the enzyme [7]. Resumption of forward SDH activity upon reperfusion leads to rapid oxidation of accumulated succinate, a process coupled with reverse electron transfer from mitochondrial complex II to complex I and reactive oxygen species production by the latter, thus contributing to cell death [7]. Interestingly, preventing succinate accumulation during ischemia using the competitive inhibitor malonate, given before transient coronary occlusion, has been shown to reduce infarct size in mice [7]. Furthermore, malonate was demonstrated to be also effective against reperfusion injury, as it was able to reduce infarct size, when given at the onset of blood flow restoration, both in isolated mice hearts and in *pigs* submitted to transient myocardial ischemia [8,12].

Emerging evidence suggests that multitargeted approaches using a combination of therapies directed against several of the intracellular signaling pathways that are involved in reperfusion injury would be helpful to reduce infarct size in the clinical setting [13]. Indeed, some combination therapies have been previously demonstrated to exert additive effects against reperfusion injury in in situ *pig* hearts, as is the case of treatments targeting cardiac metabolism and remote ischemic conditioning (RIC) [14,15]. RIC is a non-pharmacological and safe maneuver, consisting of brief episodes of ischemia and reperfusion applied remotely, mainly to a limb, that activates a variety of endogenous mechanisms of cardioprotection [16,17,18], and has been reported to exert protective effects in both experimental models [14,19,20] and in proof-of-concept clinical trials in STEMI patients [21,22]. However, the effects of a combination therapy with malonate and RIC have not been previously explored.

Thus, in this study we investigated the potential usefulness of the combination of RIC, applied during myocardial ischemia, with the SDH inhibitor malonate, given at the onset of reperfusion, to attenuate reperfusion injury and reduce infarct size in *pigs* submitted to transient coronary occlusion. Furthermore, because part of the succinate that accumulates during ischemia is released into the circulation during reperfusion [23,24,25], we also aimed to explore whether protective maneuvers may influence plasma levels of this and other citric acid cycle metabolites, with the ultimate goal of identifying a metabolic signature that may have prognostic value in the context of myocardial ischemia-reperfusion injury.

## 2. Results

### 2.1. Ischemia-Reperfusion Injury

**Figure 1:**
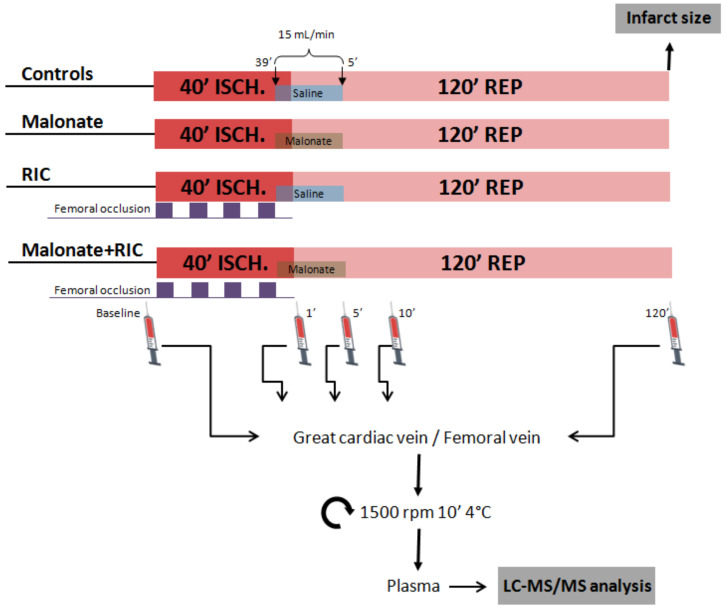
Study design. Open-chest *pigs* were submitted to 40 min of left anterior descending coronary artery occlusion followed by reperfusion and were treated with either malonate 10 mmol/L during the first 5 min of reperfusion, with 4 cycles of femoral artery occlusion (5 min each) followed by reperfusion (5 min each), applied during myocardial ischemia, or with a combination of both treatments. At the end of the experiment, infarct size was analyzed by TTC staining. Targeted LC-MS/MS analysis was performed in blood samples obtained from the great cardiac vein and a femoral vein.

Ischemia-reperfusion injury after transient coronary occlusion was assessed in control *pigs* and in animals treated with either malonate 10 mmol/L, RIC or a combination of both treatments, as depicted in Figure 1.

#### 2.1.1. Hemodynamic Variables, LAD Coronary Blood Flow and Regional Myocardial Contractility

**Figure 2:**
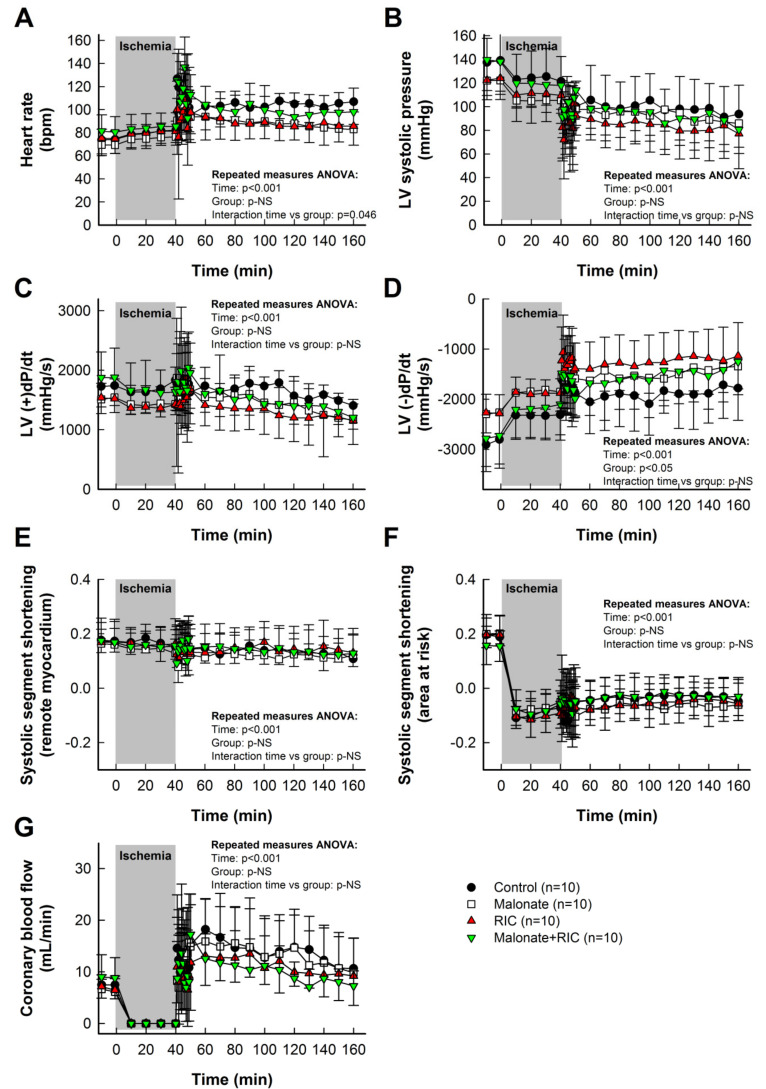
Effects of intracoronary malonate, given at the onset of reperfusion, remote ischemic conditioning (RIC), or combined treatment, on heart rate (**A**), left ventricular (LV) systolic pressure (**B**), LV (+)dP/dt (**C**), LV (−)dP/dt (**D**), systolic segment shortening in remote myocardium (**E**) and area at risk (**F**), and coronary blood flow (**G**) in pigs submitted to 40 min of LAD coronary artery occlusion followed by 2 h of reperfusion. No differences between groups were observed in the time course of any variable.

Baseline values for heart rate, hemodynamics, coronary blood flow and systolic segment shortening are shown in Table 1. As expected, ischemia-reperfusion induced significant reductions in aortic pressure, LV systolic pressure and LV (+)dP/dt, and increases in heart rate and LV (−)dP/dt, whereas coronary blood flow depicted a clear hyperemic reaction during initial reperfusion. However, the time course of these changes was similar in all experimental groups (Table 1, Figure 2).

Systolic segment shortening in distant, control myocardium, assessed by ultrasonic piezoelectric crystals, was only slightly reduced during ischemia-reperfusion (Table 1, Figure 2E). In contrast, myocardial function in the area at risk was markedly depressed during ischemia, with no recovery during reperfusion (Table 1, Figure 2F). Neither intracoronary malonate, RIC, nor combined treatment, led to an improvement in myocardial function in this region throughout the 2 h reperfusion period (Table 1, Figure 2F).

#### 2.1.2. Infarct Size

**Figure 3:**
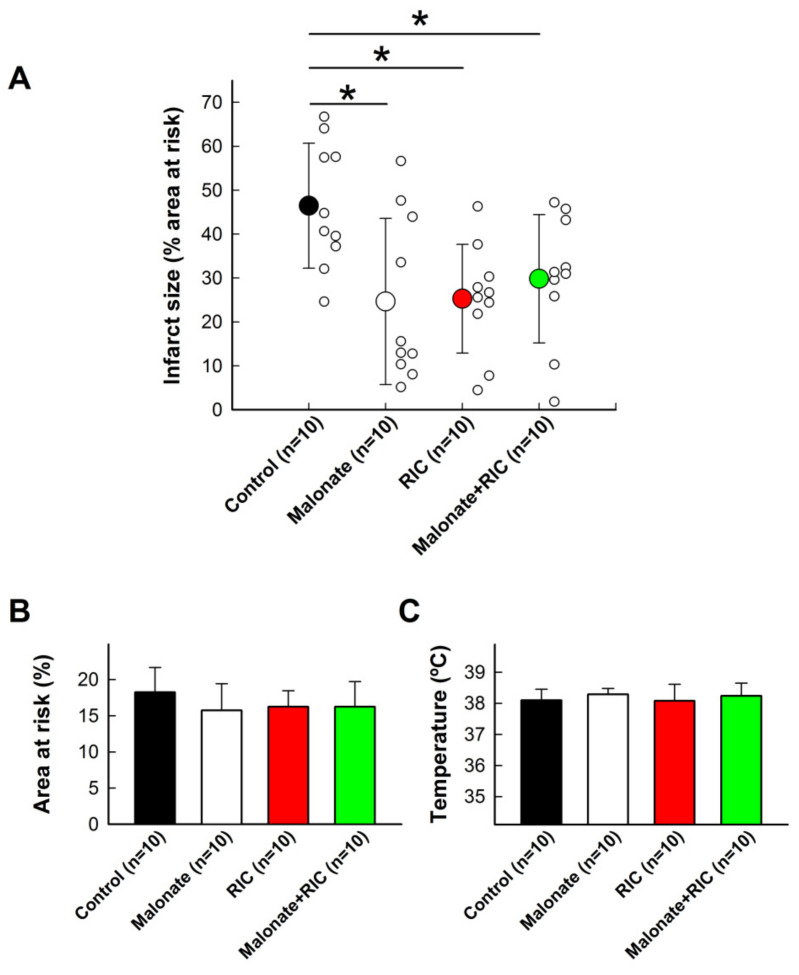
Effects of intracoronary malonate (white symbols or column), given at the onset of reperfusion, remote ischemic conditioning (RIC, red), or combined treatment (green), on infarct size (**A**) in pigs submitted to 40 min of LAD coronary artery occlusion followed by 2 h of reperfusion. Controls are shown in black. * (*p* < 0.05) indicates significant differences vs. control animals (*n* = 10/group). No differences were observed in the size of area at risk (**B**) or body temperature (**C**).

Control *pigs* submitted to 40 min of LAD coronary artery occlusion followed by reperfusion had an averaged infarct size of 46.47 ± 14.21% of the area at risk, which was significantly reduced by both 10 mmol/L of intracoronary malonate, given during initial reperfusion, and RIC (Figure 3A). However, combined treatment did not induce any additive effect, the infarct size being similar to individual treatments (Figure 3A). No differences were observed between experimental groups in the size of the area at risk or body temperature during ischemia (Figure 3B,C).

#### 2.1.3. Reperfusion Arrhythmias

**Figure 4:**
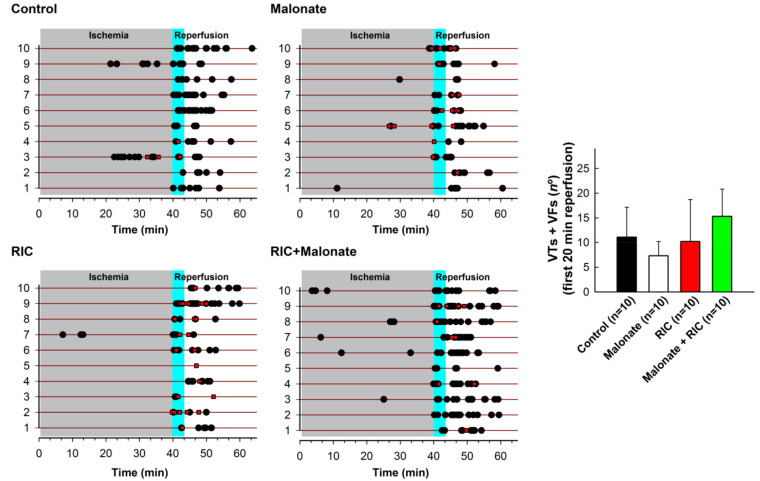
Incidence of ventricular tachyarrhythmias (ventricular tachycardias (VTs, black dots) and ventricular fibrillations (VFs, red squares) during ischemia and the first 20 min of reperfusion in control *pigs* submitted to 40 min LAD coronary occlusion followed by reperfusion, and in animals treated with intracoronary malonate, given at the onset of reperfusion, remote ischemic conditioning (RIC), or combined treatment. Right figure indicates the total number of ventricular tachyarrhythmias.

No significant differences were observed in the total number of ventricular tachyarrhythmias (ventricular tachycardias (VT) + ventricular fibrillations (VF)) during the first 20 min of reperfusion (Figure 4). However, the incidence of ventricular fibrillation during initial reperfusion was significantly higher in animals treated with malonate (8 out of 10 animals developed FV) and RIC (10 out of 10) as compared with controls (2 out of 10, *p* < 0.01) (Figure 4). This enhancement was associated with a trend towards a lower number of ventricular tachycardias in these two groups, which reaches significance in malonate-treated *pigs* (*p* < 0.05).

### 2.2. Targeted LC-MS/MS Analysis of Citric Acid Cycle Metabolites in Porcine Plasma Samples 

**Figure 5:**
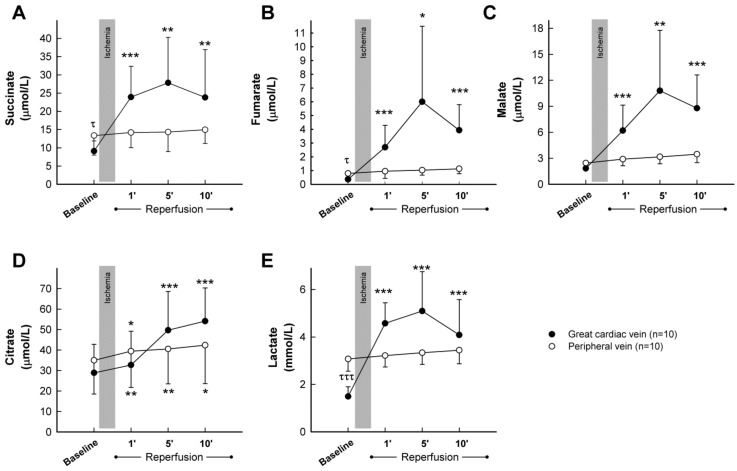
Concentrations of the citric acid cycle metabolites succinate (**A**), fumarate (**B**), malate (**C**), and citrate (**D**), together with lactate (**E**), assessed by LC-MS/MS, in plasma samples obtained from the great cardiac and femoral veins in control *pigs* submitted to 40 min of LAD coronary artery occlusion followed by 2 h of reperfusion. * (*p* < 0.05), ** (*p* < 0.01) and *** (*p* < 0.001) indicate significant differences vs. the corresponding baseline value (repeated measures ANOVA and Tukey tests). τ (*p* < 0.05) and τττ (*p* < 0.001) indicate significant differences between both baseline values (Student’s *t* test).

Analysis of peripheral blood samples obtained from control *pigs* submitted to transient coronary occlusion did not show any significant change in plasma concentrations for succinate, fumarate, malate, or lactate during the first 10 min of reperfusion (Figure 5A–C,E, blank dots), whereas a slight and continuous increase in citrate was observed in these samples following ischemia (Figure 5D, blank dots). Only modest enhancements in fumarate (from 0.80 ± 0.50 μmol/L at baseline to 1.97 ± 1.93 at the end of the experiment, Student’s *t* test, *p* = 0.039) and malate (from 2.45 ± 0.75 μmol/L to 4.89 ± 3.49, Student’s *t* test, *p* = 0.031) were noticeable 2 h after reperfusion. In contrast, plasma samples obtained from the great cardiac vein depicted, in this group of animals, a marked increase in all analyzed metabolites, peaking, in most cases, at 5 min of reperfusion (Figure 5A–E, black dots). Differences in baseline values between peripheral and great cardiac vein samples were apparent for succinate, fumarate, and especially, lactate. Malonate was not detected in these animals at any time.

**Figure 6:**
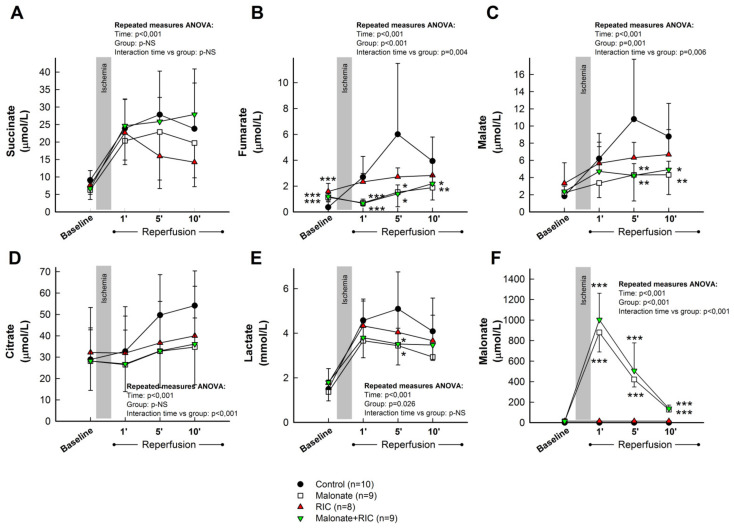
Concentrations of the citric acid cycle metabolites succinate (**A**), fumarate (**B**), malate (**C**) and citrate (**D**), together with lactate (**E**) and malonate (**F**), assessed by LC-MS/MS, in plasma samples obtained from the great cardiac vein in control *pigs*, submitted to 40 min of LAD coronary artery occlusion followed by 2 h of reperfusion, and in animals treated with intracoronary malonate, given at the onset of reperfusion, remote ischemic conditioning (RIC), or malonate + RIC. * (*p* < 0.05), ** (*p* < 0.01) and *** (*p* < 0.001) indicate significant differences vs. the corresponding value in the control group (ANOVA and Tukey tests).

As these results demonstrate that citric acid cycle metabolites are only enhanced in plasma samples obtained from the great cardiac vein, we decided to assess the influence of malonate, RIC, or combined treatment only in blood from this source. Accordingly, repeated measures ANOVA analysis demonstrated significantly enhanced levels of succinate during reperfusion in all experimental groups, with no differences between treatments (Figure 6A). In contrast, the increase in fumarate and malate detected during initial reperfusion was significantly attenuated by cardioprotective maneuvers. Indeed, repeated measures ANOVA demonstrated a significant effect of group allocation for both fumarate and malate, together with positive interactions between group allocation and the time course of concentration changes (Figure 6B,C). In addition, the enhancement in citrate levels observed in control animals was also attenuated in all treated groups (Figure 6D), whereas a marginally significant effect of group allocation was detected for lactate (Figure 6E). Measurement of malonate, used as a positive control, demonstrated an increase in its plasma concentration only in animals treated with this reversible SDH inhibitor (Figure 6F).

When analyzed independently of the control group, pooled samples from the two groups receiving malonate showed a trend of having higher succinate concentrations than those receiving RIC alone (repeated measures ANOVA, *p* = 0.051 for interaction), and significantly reduced levels of fumarate (repeated measures ANOVA, *p* = 0.014 for interaction and *p* < 0.001 for group allocation) and malate (repeated measures ANOVA, *p* = 0.019 for group allocation).

**Figure 7:**
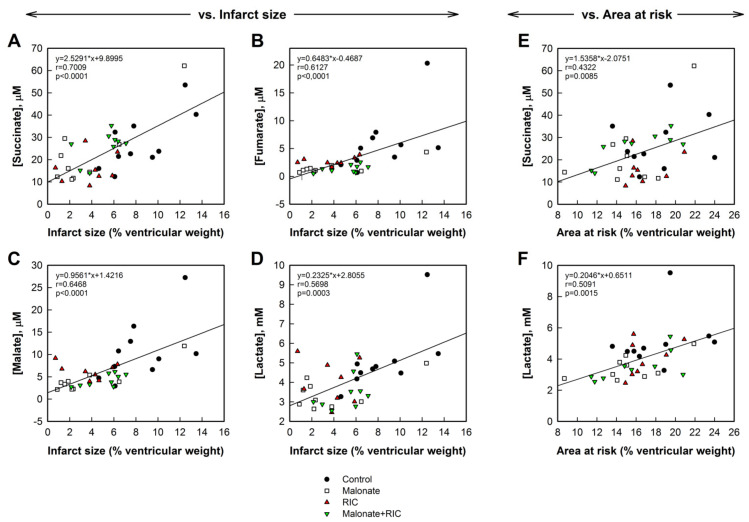
Correlations between infarct size, expressed as percentage of total cardiac weight, and succinate (**A**), fumarate (**B**), malate (**C**), and lactate (**D**) concentrations measured 5 min after reperfusion in plasma obtained from the great cardiac vein in *pigs* from the four experimental groups. Right panels show correlations between area at risk (in percentage of ventricular weight) and succinate (**E**) and lactate (**F**) concentrations in the same plasma samples.

Interestingly, concentrations of succinate, fumarate, malate, and lactate, obtained 5 min after reperfusion from the great cardiac vein, significantly correlated with infarct size, expressed as percentage of ventricular weight, with good correlation coefficients, particularly for succinate levels (Figure 7A–D). Similar data were obtained when infarct size was expressed in grams (not shown). In contrast, only succinate and lactate concentrations correlated with the size of the area at risk, although in these cases correlation coefficients were slightly lower (Figure 7E,F). No group differences were observed in these correlationships.

## 3. Discussion

This study demonstrates that the combination of SDH inhibition during initial reperfusion with intracoronary malonate and RIC exerts non-additive cardioprotective effects in *pigs* submitted to transient coronary occlusion. Moreover, our results show that the enhanced release of citric acid cycle intermediates to the bloodstream, detected in plasma samples from the great cardiac vein during initial reperfusion, is attenuated by cardioprotective maneuvers, and that succinate, fumarate, and malate concentrations significantly correlate with infarct size. These results open up the possibility to use citric acid cycle metabolite concentrations obtained in blood samples from the coronary sinus as new prognosis biomarkers in STEMI patients.

### 3.1. Cardioprotection by SDH Inhibition and RIC

Prevention of succinate accumulation during ischemia by pretreatment with the reversible SDH inhibitor malonate was demonstrated to reduce myocardial infarct size in several animal models [7,26,27]. Although the mechanisms of succinate accumulation in ischemic tissues are not entirely understood [7,24,28], it was demonstrated that accumulated succinate is rapidly oxidized during reperfusion by forward SDH activity, which, in turn, induces a massive reactive oxygen species (ROS) production by reverse electron transfer from mitochondrial complex II to complex I [7,8]. This oxidative stress is associated with mitochondrial permeability transition pore (MPTP) opening and cell death [7,8]. Furthermore, it has been demonstrated that administration of the SDH inhibitor malonate at the onset of reperfusion reduces myocardial infarct size in several independent experimental models, including Langendorff-perfused isolated mice heart [8], in situ coronary occlusion in mice [26], and in *pigs* submitted to transient coronary occlusion [12]. In all of these models, the protective effect of malonate was associated with reduced oxidation of succinate during the first minutes of reperfusion, less ROS production, preserved mitochondrial function, and increased calcein retention in isolated mitochondria, suggestive of reduced MPTP opening [8]. Our present results thus support the cardioprotective effects of SDH inhibition when applied at the onset of reperfusion.

RIC activates a variety of endogenous mechanisms of cardioprotection [16,17,18] and has been shown to exert protective effects in both experimental models [14,19,20], and in proof-of-concept clinical trials in STEMI patients [21,22]. Experimental studies have demonstrated that the cardioprotective signal is transferred from the remote conditioned organ or tissue to the heart, either through the release of humoral factors or through activation of neuronal pathways [16]. In the heart, RIC causes activation of intracellular transduction cascades similar to those of classic, local, ischemic pre- or postconditioning, including the RISK and SAFE pathways [16,17,29], being the mitochondria the end-effectors [16,17,30]. Our present results confirm previous data obtained by our group in the same animal model [14,15], and support the cardioprotective effect of RIC against myocardial infarction. Unfortunately, however, two recently published randomized clinical trials have not been able to find any effect of RIC on clinical outcomes or on infarct size evaluated by cardiac biomarkers or NMR in STEMI patients [31,32]. Different reasons have been proposed to explain the failure of translation of cardioprotective strategies, including presence of comorbidites, such as aging, diabetes, or hypertension, which may alter the efficacy of cardioprotective maneuvers [33], the routine use of different comedications, such as P2Y12 antagonists, which may have protective effects [34], or the lack of uniformity in the method used to quantify infarct size [32]. Optimization of conditioning protocols in the clinical setting may help to improve efficacy of RIC in STEMI patients [32].

Under this situation, emerging evidence suggests that a multitargeted approach using two or more therapies, directed against different signaling pathways or cell targets involved in ischemia-reperfusion injury, would be advantageous in STEMI patients in order to reduce final infarct size [13]. Additive protection may be reached when the mechanisms of action of the combined interventions are different and would be especially interesting when the efficacy of the individual treatments is expected to be reduced by comorbidities or comedications. To date, some of the combined strategies that have demonstrated additive protection are addressed to different cell targets, such as coronary circulation or microvascular obstruction vs. cardiomyocytes [35]. This is the case of the combination of the antiplatelet cangrelor (a P2Y_12_ receptor antagonist) and cariporide (a Na^+^/H^+^ exchanger inhibitor) or hypothermia, the last two acting mostly on cardiomyocytes, which has been shown to exert additive protection in open-chest rats submitted to transient coronary occlusion [36]. Other strategies have used treatment combinations acting on different signaling pathways within cardiomyocytes, including those targeting ischemic and reperfusion injury separately. In this regard, additive protection was found between RIC and local ischemic postconditioning in an in vivo rat model of ischemia-reperfusion [37], a finding that was later extended to STEMI patients [38]. Similarly, we have previously demonstrated additive effects between treatments modulating myocardial energy metabolism (glucose-insulin-potassium (GIK) or exenatide, a mimetic of the incretin glucagon-like peptide-1 (GLP-1)) and RIC [14,15]. Furthermore, synergistic effects between inhibition of succinate accumulation, oxidation, and hypothermia have been also described in rabbits [39]. However, not all treatment combinations are equally effective. Thus, protection by long-term nitroglycerine and RIC was shown to be abrogated when both individual treatments were combined in rats [40]. Similarly, no additive effects were found between aminooxyacetate, a malate-aspartate shuttle inhibitor, and local ischemic preconditioning in isolated rat hearts [41]. Unfortunately, our present results demonstrate that the addition of the SDH inhibitor malonate to RIC does not exert additive cardioprotective effects in *pigs* submitted to transient coronary occlusion.

As conduction arrhythmias may severely affect cardiac performance during reperfusion, we assessed whether our two individual treatments and the combination of both was able to modify the incidence of ventricular tachyarrhythmias during initial reperfusion. Accordingly, we observed that none of the three treatments were able to modify the total number of ventricular tachyarrhythmias following ischemia. However, the incidence of VF during initial reperfusion was significantly higher in animals treated with malonate and RIC as compared with controls, although this enhancement was, in part, compensated with a trend towards a lower number of VTs in these two groups. In contrast to these findings, we have previously demonstrated that neither malonate [12], nor RIC [15], were able to modify the incidence of VFs in the same animal model. Reasons for these discrepancies are unknown. It is plausible that smaller, patchier, infactions in malonate and RIC-treated animals in the present study would create the ideal substrate for VF to evolve, at the expense of a lower number of VTs. Nevertheless, the higher incidence of VFs in these two groups in the present study would not modify our main conclusion, as defibrillation has been associated with higher infarctions in the *pig* model [42]. Thus, even smaller infactions would be expected in these two groups in the absence of VF, thus magnifying its protective action. On the contrary, the incidence of VF in *pigs* receiving the treatment combination was not modified, thus supporting the fact that both treatments have no additive effect on infarct size.

### 3.2. Targeted Analysis of Citric Acid Cycle Metabolites by LC-MS/MS

Part of the succinate that accumulated during myocardial ischemia is released into the bloodstream after flow restoration [23,24,25]. In fact, conditions during early reperfusion might not be so favorable for reverse electron transfer, and the rapid decay in succinate occurring during reperfusion [7,8] has been suggested to be due, at least in part, to efflux rather than oxidation [43]. Indeed, it has been quantified that about two-thirds of accumulated succinate is washed into the perfusate within the first 5 min of reperfusion, while approximately one-third is metabolized [24]. Supporting the existence of this efflux, succinate, together with other citric acid cycle metabolites, including fumarate, malate and citrate, have been found to accumulate in the interstitial space during ischemia and initial reperfusion in isolated rat hearts [41]. Similar findings were obtained in the right ventricular interstitial space of newborn *pigs* submitted to 10 cycles of 3 min ischemia followed by 3 min of reperfusion, as determined using microdialysis catheters [44]. However, analysis of interstitial metabolite concentrations in newborn animals in the last study might not be directly translated to changes occurring in adult hearts, as a gradual switch in cardiac energy generation from glycolysis to fatty acid oxidation occurs over the first postnatal weeks [44]. Furthermore, short bouts of ischemia in that study [44] may not completely reflect changes occurring after myocardial infarction. Importantly, Prag and coworkers have recently suggested that succinate release upon reperfusion of the ischemic heart is mediated by the monocarboxylate transporter 1 (MCT1), in a process that is facilitated by ischemic acidification of the myocardium [25].

Several studies have characterized the plasma metabolome in patients with STEMI. Using a non-targeted LC-MS approach in 27 STEMI patients who underwent primary percutaneous coronary intervention (pPCI), it was demonstrated that the largest cohort of molecules undergoing significant changes, 2 to 48 after pPCI, were lipid metabolites, although citric acid metabolites, and especially succinate were also increased [45]. In contrast, others found a decrease in succinate, fumarate, and citrate in serum obtained 1 h after symptom onset in 20 STEMI patients [46]. Significantly, peripheral blood might not be appropriate to analyze these changes, which could explain these discrepancies. Indeed, it was demonstrated in STEMI patients, and in a porcine model of transient coronary occlusion, that the greatest increase in succinate concentrations occurred when blood was taken, immediately after reperfusion, from the coronary sinus [23,25]. However, whether release of succinate and other citric acid cycle metabolites to the bloodstream during reperfusion is modified by protective maneuvers was until now unknown. In this sense, our present results demonstrate that the enhanced levels of fumarate, malate, and citrate, detected in the plasma from the great cardiac vein in control animals during initial reperfusion, are reduced by both individual treatments and by a combination of RIC with malonate. In addition, it was unknown whether citric acid cycle metabolite concentrations might have a prognostic value during myocardial ischemia-reperfusion. In the study by Kohlhauer et al. including STEMI and non-STEMI patients, a modest correlation between coronary sinus succinate concentrations and edema volume, but not irreversible myocardial injury (i.e., final myocardial infarct size at 6 months, as measured by cardiac magnetic resonance, or troponins, determined during the first 48 h), was found [23]. Edema volume was quantified, in the aforementioned study, by T2-weighted nuclear magnetic resonance imaging performed 2 days after pPCI, a supposed surrogate of area at risk and acute injury [23]. However, it has been suggested that T2-weighted edema might not constitute an accurate surrogate for the area at risk [47], especially when it is not determined in a time window ranging between 4 and 7 days post myocardial infarction [48] and when cardioprotective therapies are applied [49]. Our present results using a targeted LC-MS/MS approach demonstrate that not only succinate, but also fumarate and malate, are significantly enhanced during initial reperfusion in blood from the great cardiac vein, confirm a moderate correlation between succinate (and lactate) concentrations measured 5 min after reperfusion with the size of the area at risk, and suggest positive correlations between succinate, fumarate, and malate and myocardial infarct size. These data are suggestive that citric acid metabolite concentrations obtained in blood samples from the coronary sinus might constitute a new prognosis tool to predict final infarct size in STEMI patients.

The increase in citric acid cycle intermediates seen after reperfusion can be due to two different mechanisms that may work simultaneously. Membrane rupture would cause a massive release of intracellular metabolites, and in this sense, our present results may merely reflect differences in the amount of cell death. However, it may also reflect differences in the metabolic profile of cardiac cells and active release of metabolites. Accordingly, our present data demonstrate that groups receiving malonate had higher succinate plasma concentrations as compared with those receiving RIC alone. As these groups of animals have similar infarctions, these differences can only be ascribed to the effect of malonate on SDH activity [7,8]. Furthermore, these differences seem to extend to fumarate and malate.

## 4. Materials and Methods

This study complies with Directive 2010/63/EU of the European Parliament on the protection of animals used for scientific purposes and the NIH Guide for the Care and Use of Laboratory Animals (NIH publications Nº. 85-23, revised 1996, updated in 2011). The study was approved by the Ethics Committee of our institution (reference number: CEEA 33/17).

### 4.1. Animals and Instrumentation

Forty hybrid farm *pigs* (25–30 kg, 12 h fasting) were premedicated with tiletamine-zolazepam (4–6 mg/kg, IM) and anesthetized with sodium thiopental (25 mg/kg, IV, plus continuous infusion at 6–14 mg/kg/h) and fentanyl (5 μg/kg, IV, plus continuous infusion at 3–6 μg/kg/h). Following ventilation, the thorax was opened, and the left anterior descending (LAD) coronary artery was dissected free below the first diagonal branch [12,14]. Electrocardiogram, left ventricular (LV) pressure, LV dP/dt and coronary blow flow were recorded in a computer as previously described [12,14]. At the end of the experiments, animals were sacrificed by a pentobarbital overdose (100 mg/kg, IV).

### 4.2. Regional Myocardial Function

Two pairs of hemispherical polystyrene crystals were inserted into the remote LV myocardium and in the area at risk to monitor regional myocardial function, as previously described [12,14,50]. Systolic segment shortening ratio (SS) was calculated as SS = (EDL − ESL)/EDL, where EDL is end-diastolic length and ESL corresponds to end-systolic length [12,14,50].

### 4.3. Study Protocols

All animals were intravenously administered with sodium heparin (100 UI/kg). Immediately, a Judkins 8F guiding catheter was inserted into a carotid artery, and a 2.8/2.5F intracoronary infusion catheter (TRANSIT, Cordis Neurovascular Inc., Miami, FL, USA) was advanced through it into the LAD, until crossing the dissection site selected for coronary occlusion [12]. To assess the effects of treatments on ischemia-reperfusion injury, *pigs* were then submitted to 40 min of LAD coronary artery occlusion, followed by 2 h of reperfusion. This time of ischemia (40 min) was selected based on previous publications from our group that demonstrated that this duration induced an infarct size of about 50% of the area at risk [12,14]. Myocardial ischemia was performed by occluding the LAD coronary artery around the infusion catheter using an elastic snare. Animals were randomly assigned to four different experimental groups (*n* = 10/group). Control *pigs* received intracoronary saline for 6 min, beginning at 39 min of ischemia and lasting for the first 5 min of reperfusion, at a flow rate of 15 mL/min (37 °C). Malonate-treated animals received intracoronary saline containing disodium malonate at a concentration of 10 mmol/L. Conditioned animals (RIC) were submitted, in addition to intracoronary saline infusion, to four cycles of 5 min of right lower limb ischemia followed by 5 min of reperfusion, starting simultaneously with LAD ligature, using an elastic snare placed around the right femoral artery. Finally, a fourth treatment group consisted in the combination of both treatments.

Blood samples were obtained in all cases at baseline and 1, 5, and 10 min after the onset of reperfusion, both from the great cardiac vein (or anterior interventricular vein), running parallel to the LAD coronary artery, and from the left femoral vein. An additional peripheral blood sample was obtained at the end of reperfusion (2 h). Blood was collected in heparinized tubes, centrifuged at 1500 g for 10 min (4 °C) to obtain plasma, and stored at −80 °C until analysis.

### 4.4. Area at Risk and Infarct Size

Two hours after reperfusion, the LAD was reoccluded and the size of the area at risk and of infarction were determined by 10% fluorescein and 1% 2,3,5-triphenyltetrazolium chloride (TTC) staining, respectively, as previously described [51]. Area at risk was expressed as percentage of total ventricular weight and infarct size as percentage of area at risk.

### 4.5. Reperfusion Arrhythmias

Recordings were analyzed for the incidence of ventricular tachycardia (VT) and ventricular fibrillation (VF) during ischemia and the first 20 min of reperfusion. VT was defined as three or more consecutive premature beats of ventricular origin at a heart rate faster than 120 beats/min, and wide QRS durations (>120 ms) [15].

### 4.6. Targeted LC-MS/MS Analysis of Citric Acid Cycle Metabolites in Porcine Plasma Samples

Separation and detection of the citric acid cycle metabolites succinate, fumarate, malate, and citrate, together with lactate and malonate, was performed on a Waters Acquity Ultra Performance Liquid Chromatographic coupled with a Waters Xevo TQ MS triple quadrupole mass spectrometer (Waters Corporation, Milford, MA, USA).

Standard calibration curves for each analyte were freshly prepared by adding 5 µL of 7 different concentrations of the distinct metabolites to 200 μL of plasma. An additional blank sample lacking analytes was also prepared. Linearity for the standard calibration curves was obtained between 3.125 and 500 μmol/L for succinate, 0.1 and 20 μmol/L for fumarate, 0.5 and 40 μmol/L for malate, 7.8125 and 125 μmol/L for citrate, 0.005 and 2500 μmol/L for malonate, and 500 and 8000 μmol/L for lactate. Metabolites were then extracted by adding 200 μL or 400 μL of methanol, containing 0.05 mmol/L succinic acid-2,2,3,3-d_4_ (#293075, Merck KGaA, Darmstadt, Germany) as an internal standard, to 100 μL of each sample or 200 μL of each calibration standard, respectively. Samples were vortexed, cooled at −20 °C for 20 min, and centrifuged at 11,000 rpm for 30 min, at 4 °C. Supernatants containing metabolites were transferred to new 1.5 mL propylene tubes, lyophilized, and stored at −20 °C until analysis. Before injection to LC-MS/MS, the purified residues were reconstituted with 100 μL (for samples) or 200 μL (for calibration standards) of a mobile phase solution consisting of mobile phase A (0.2% formic acid in acetonitrile) and mobile phase B (0.2% formic acid in water) at 10:90 *v*/*v*. Samples and calibration standards were vortexed for 5 min and centrifuged again at 11,000 rpm for 5 min, at 4 °C.

Separation was achieved following injection of 4 μL of each sample on an Acquity UPLC HSS C18 column (2.1 × 100 mm, 1.8 μm particle size, Waters Corporation, Milford, MA, USA). A gradient elution program was conducted for chromatographic separation with mobile phase A and mobile phase B as follows: 0–1.5 min hold for 10% eluent A, 1.5–5 min from 95% to 60% eluent A, 5–7 min hold for 60% eluent A, 7–7.5 min from 60% to 10% eluent A and 7.5–9 min hold for 10% eluent A to reequilibrate column before next injection. Pump was operated at a flow rate of 0.3 mL/min with an overall run time of 9 min. The autosampler was held at 6 °C and column oven was set up at 30 °C. The mass spectrometer was operated in multiple reaction monitoring (MRM) using an electrospray (ESI) source in negative mode for all compounds, with a capillary voltage of 2.02 kV. Argon was used as collision gas and flow was 0.17 mL/min. Desolvation temperature was 450 °C with a gas flow of 1100 L/h. Ion transitions and optimal cone voltage and collision energy use for fragments detection are summarized in Table 2. System control and data analysis were carried out using the MassLynx software (Version 4.1, Waters Corporation, Milford, MA, USA) and processed using TargetLynxTM program (Waters Corporation, Milford, MA, USA). For each calibration standard, the ratio between the intermediate peak and the internal standard containing succinic acid-2,2,3,3-d_4_ was determined. Linear regressions describing the calibration curves were then calculated using a weighting factor of 1/x^2^, where x was concentration.

### 4.7. Statistical Analysis

All measurements were carried out by a researcher blinded to group allocation. Normal distribution was assessed by Kolmogorov–Smirnov test. Data are expressed as mean±SD. ANOVA and Tukey post-hoc test were used to assess differences in infarct size and area at risk. Changes in the time course of hemodynamic and contractility variables and in metabolomic studies were assessed by repeated measures ANOVA and Tukey post-hoc tests. Student’s t test was used to compare metabolite concentrations at a baseline between peripheral and great cardiac vein plasma samples. Non-parametric Kruskal–Wallis test was used to assess differences in the number of ventricular tachyarrhythmias. Incidence of VF was analyzed by the Pearson Chi-square test. Differences were considered significant when *p* < 0.05.

## 5. Conclusions

Our present results demonstrate that, despite the combination of SDH inhibition during reperfusion and RIC did not result in additive protection, plasma concentrations of selected citric acid cycle metabolites are attenuated by protective maneuvers and correlate with irreversible injury. Analysis of these metabolites may, therefore, have a prognostic value in STEMI patients.

## Figures and Tables

**Table 1:** Values at baseline and at the end of ischemia (40 min) and reperfusion (2 h) for heart rate, aortic pressure, left ventricular (LV) systolic and end-diastolic pressure, LV (+) and (−)dP/dt, coronary blood flow at the LAD, and systolic shortening (SS) in the control and ischemic areas, in control *pigs* and in *pigs* receiving intracoronary malonate, remote ischemic conditioning (RIC), or malonate + RIC. Data are shown as mean ± SD.

		Heart Rate (Beats/min)	Aortic Pressure (mm Hg)	LV Systolic Pressure (mm Hg)	LV End-Diastolic Pressure (mm Hg)	LV (+)dP/dt (mm Hg/s)	LV (–)dP/dt (mm Hg/s)	LAD Coronary Blood Flow (mL/min)	SS (Control)	SS (Ischemic)
Control	Baseline	74.17 ± 12.29	114.97 ± 14.73	139.14 ± 12.59	6.09 ± 3.242	1743 ± 337	−2803 ± 491	7.63 ± 1.72	0.17 ± 0.04	0.19 ± 0.08
(*n* = 10)	40′ isch	82.24 ± 13.06 *	101.97 ±1 3.67 *	121.39 ± 21.01 *	5.51 ± 4.07	1834 ± 506	−2312 ± 507 *	0.00 ± 0.00 *	0.15 ± 0.05	−0.07 ± 0.02 *
	2 h reperf	106.86 ± 19.70 *	81.39 ± 20.35 *	93.72 ± 24.52 *	4.21 ± 3.57	1407 ± 397 *	−1778 ± 644 *	8.18 ± 5.83	0.11 ± 0.03 *	−0.05 ± 0.08 *
Malonate	Baseline	69.35 ± 12.21	104.76 ± 10.60	122.17 ± 16.18	4.28 ± 3.33 τ	1543 ± 310	–2263 ± 431	6.12 ± 2.57	0.16 ± 0.06	0.20 ± 0.05
(*n* = 10)	40′ isch	76.46 ± 13.54 *	90.38 ± 14.69 *	105.36 ± 19.52 *	3.81 ± 4.06	1432 ± 282 *	−1852 ± 387 *	0.00 ± 0.00 *	0.13 ± 0.05	−0.05 ± 0.05 *
	2 h reperf	82.98 ± 14.95 *	76.64 ± 23.35 *	85.90 ± 25.49 *	3.06 ± 3.50	1164 ± 346 *	−1347 ± 563 *	9.79 ± 6.76	0.12 ± 0.07	−0.06 ± 0.06 *
RIC	Baseline	74.85 ± 12.72	102.56 ± 8.74	124.06 ± 13.61	8.20 ± 1.29 τ	1522 ± 232	−2283 ± 374	6.45 ± 1.67	0.17 ± 0.08	0.20 ± 0.07
(*n* = 10)	40′ isch	81.35 ± 9.84	93.19 ± 7.42 *	109.55 ± 11.73 *	7.11 ± 1.72	1428 ± 175	−1878 ± 286 *	0.00 ± 0.00 *	0.16 ± 0.05	−0.09 ± 0.08 *
	2 h reperf	85.66 ± 16.47	63.95 ± 23.00 *	77.17 ± 29.59 *	4.85 ± 2.11 *	1152 ± 401 *	−1137 ± 664 *	9.19 ± 5.69	0.13 ± 0.09	−0.06 ± 0.08 *
Malonate	Baseline	80.59 ± 13.34	112.80 ± 23.53	138.18 ± 25.03	5.13 ± 2.52	1879 ± 492	−2730 ± 646	8.82 ± 3.93	0.17 ± 0.08	0.16 ± 0.06
+ RIC	40′ isch	85.40 ± 11.96	98.16 ± 18.86 *	117.95 ± 23.52 *	5.09 ± 4.93	1636 ± 415 *	−2102 ± 611 *	0.00 ± 0.00 *	0.16 ± 0.10	−0.06 ± 0.09 *
(*n* = 10)	2 h reperf	98.10 ± 20.61	69.41 ± 14.26 *	80.62 ± 14.91 *	3.77 ± 3.06	1204 ± 229 *	−1254 ± 539 *	7.27 ± 2.91	0.13 ± 0.07	−0.03 ± 0.07 *

* (*p* < 0.05) indicates significant differences vs. the corresponding baseline value (repeated measures ANOVA); τ (*p* < 0.05) indicates significant differences between malonate and RIC baseline values (ANOVA and Tukey tests).

**Table 2:** Ion transitions and optimal cone voltage and collision energy used for fragments detection in LC-MS/MS analysis of citric acid cycle metabolites in porcine plasma samples. IS indicates internal standard.

Compound	Precursor Ion m/z	Product Ion	Cone Voltage	Collision Energy
Succinate	117.10	73.00	16	11
Succinic acid-2,2,3,3-d_4_ (IS)	121.10	76.50	15	9
Fumarate	115.09	71.01	13	6
Malate	133.04	115.02	20	10
Citrate	191.13	111.00	18	10
Malonate	103.09	59.00	12	8
Lactate	89.11	43.00	18	10

## Data Availability

The data presented in this study are available on request from the corresponding author.
